# In-vitro osteoblast proliferation and in-vivo anti-osteoporotic activity of *Bombax ceiba* with quantification of Lupeol, gallic acid and β-sitosterol by HPTLC and HPLC

**DOI:** 10.1186/s12906-018-2299-1

**Published:** 2018-08-07

**Authors:** Shashi Chauhan, Aditi Sharma, Navneet Kumar Upadhyay, Gajender Singh, Uma Ranjan Lal, Rohit Goyal

**Affiliations:** School of Pharmaceutical Sciences, Shoolini University, Solan, HP 173212 India

**Keywords:** *Bombax ceiba*, Lupeol, Gallic acid, Ovariectomy, Osteoporosis, Estrogen

## Abstract

**Background:**

*Bombax ceiba* is used traditionally to treat bone disorders, rheumatism, and joint pain. The aim of the study is to carry out osteogenic activity in-vitro and anti-osteoporotic activity in-vivo of stem bark of *B. ceiba* in surgical ovariectomy model in female rats.

**Methods:**

Plant drug: *B. ceiba* stem bark was extracted with solvents petroleum ether and methanol using Soxhlet extraction. In-vitro osteoblastic proliferation study was performed using UMR-106 cell lines. Both the extracts were undergone to acute toxicity study as per OECD423 guidelines. Female Wistar albino rats 180-240 g were used (*n* = 6). Surgical ovariectomy was performed under anesthesia to induce bone porosity and loss in all animals except normal control and sham control. Each extract was administered at two dose level: 100 and 200 mg/kg and the standard Raloxifene was given at 1 mg/kg orally for 28 days. The phytochemical study of both the extracts was performed using HPLC and HPTLC.

**Results:**

A significant osteoblast cell proliferation and alkaline phosphatase activity were observed with *B. ceiba* extracts in UMR-106 cell lines. Surgical removal of ovaries produced significant (*p* < 0.05) decline in bone mineral density, bone breaking strength, serum ALP, calcium, phosphorus, and estradiol level and marked bone tissue destruction in histology. Administration of petroleum ether and methanolic extract for 28 days significantly (*p* < 0.05) ameliorated the consequences of ovariectomy induced bone porosity and restored the normal architecture of bone, as compared to OVX control. The phytochemical screening of both the extracts were also carried out. The quantification of phytoconstituents showed the presence of β-sitosterol and lupeol in petroleum ether extract, whereas the lupeol is also quantified in the methanolic extract. The presence of gallic acid was quantified in methanolic extract using HPLC.

**Conclusion:**

*B. ceiba:* stem bark ameliorated the state of bone fragility and fracture possibly due to estrogenic modulation, as also confirmed by in-vitro osteogenic activity which may be due to the presence of lupeol, gallic acid and β-sitosterol constituents of the plant.

## Background

Osteoporosis is a progressive, chronic bone disorder of metabolic or hormonal dysregulation and revealed by severe bone porosity, fractures and low bone mass due to microarchitectural deterioration. It occurs due to alteration in the processes of bone tissue formation and resorption, which are characterized by low mechanical bone strength and increased bone fracture [1, 2]. The epidemiology of the debilitating state depicts that approximately 75 million people suffer from bone fragility and other bone disorders in the United States, Europe and Japan [3]. The World Health Organization states that 30% of postmenopausal women have incidences of bone fractures of the pelvis and lumbar spine or distal forearm [4]. The modern medication suggested to date are calcium and vitamin D supplements, bisphosphonates, selective estrogen receptor modulators, hormone replacement therapy, calcitonin, parathyroid hormone, monoclonal antibodies, and a recombinant form of bone morphogenic protein (BMP)-2 and − 6. But these medications for long-term use cause severe adverse effects like hypercalciuria, hypercalcemia, musculoskeletal pain, osteonecrosis of the jaw, breast tenderness, thromboembolic events, and increased risk of endometrial and breast cancer [5].

Bone remodeling refers to the process of bone formation and sorption of activation of osteoblast and osteoclasts cells respectively. In postmenopausal aged women, the lack of gonadal hormone: estrogen is noted which results in osteoclastogenesis and bone loss. In numerous studies, the state of postmenopausal osteoporosis and estrogen deficiency in women are evaluated by surgical ovariectomy model in female rats. Receptor-activated for Nuclear Kappa (RANK) is expressed in osteoblast progenitors and T lymphocytes [6–8]. RANK ligand (RANKL) binds to a RANK receptor expressed on osteoclast, causes activation and production of pro-inflammatory mediators: TNF-α, IL-1 & IL-7, and downregulation of osteoprotegerin (OPG) [9]. Physiologically, the expression of fatty acid synthase (FAS) gene is also increased due to a deficiency of estrogen [10, 11]. FAS is an enzyme capable of de novo long chain fatty acid synthesis [12]. In an in-vitro study, suppression of fatty acid biosynthesis using FAS inhibitor is reported to prevent lipotoxicity in adipocyte on human osteoblasts [13]. In an in-vitro study, co-culture mature adipocytes showed decreased osteoblasts proliferation [14]. The estrogen regulates activation of BMP-2 and transforming growth factor (TGF-β) in osteoblasts [15]. TGF-β antagonizes the glucocorticoids induced FAS upregulation [16], and BMP-2 is also reported to suppresses FAS expression [17]. UMR-106 cell has several phenotypic properties matures to osteoblast cells. They appear morphological appearance with calciotropic agents and an expression of alkaline phosphatase as a marker of osteoblastic differentiation, mineralization and bone formation [18, 19].

*B. cieba* L. belongs to the family: Bombacaceae is a deciduous tall tree with typical woody spines on the branches and trunk [20]. Stem bark of the plant is reported to contain lupeol, β-sitosterol, shamimicin [21], ceibanephthaquinone, silamiln-A and silamalin-B [22], magniferin, epicatechin-3-O-b-xylopyra-noside, epicatechin-7-O-b-xylopyranosid [23], shamiminol, stigmasta-3,5-diene, lupenone and opuntiol [24]. The plant is reported as anti-inflammatory, anti-oxidant, anti-obesity, hepatoprotective, hypoglycemic and hypotensive activities [25, 26]. *B. ceiba* is used for inflammatory diseases such as asthma, diabetes, wounded glandular swelling [27]. Ethnopharmacologically, it is primarily applied to treat dislocated bones, rheumatism and body pain [28]. The quantification of a flavonoid, quercetin was also reported from the leaves of *B. ceiba* [29]. Dietary form of quercetin inhibits loss of bone integrity cause due to ovariectomy in mice [30] and documented for bone repair properties [31].

The setting of the study is coupled with the efficacy of Lupeol, Gallic acid, and β-sitosterol possibly in bone remodeling and hence the present work was hypothesized to investigate the pharmacological activity of *B. ceiba* stem bark extract against the altered state of bone remodeling in surgical ovariectomy-induced osteoporosis in female rats and phytochemical evaluation of *B. ceiba* extract by HPTLC and HPLC.

## Methods

### Plant drug extraction and qualitative phytochemical screening

The stem bark of *B. ceiba* was collected from Sunder Nagar, HP, India and authenticated from National Institute of Science Communication and Information Resources, New Delhi and a specimen was linked to the institutional herbarium (NISCAIR/RHMD/Consult/11–12/1758/58). Plant part was shade dried, powdered coarsely up to sieve mesh size 10 and stored in an airtight container till further use. Plant drug was extracted with Soxhlet extractor using solvents like petroleum ether and methanol in ratio 1:3 *w*/*v* for 48 h. The solvent was slowly distilled, the concentrate was dried *in-vacuo* and kept in air-tight containers, desiccator. The phytochemical screening of prepared petroleum ether and methanolic extract of *B. ceiba* was carried out to investigate the phytoconstituents like steroids, glycosides, flavonoids, tannins, phenolic, terpenoids, proteins/amino acids, lipids/fixed oil and carbohydrates qualitatively [32, 33].

### Quantification of gallic acid using high-performance liquid chromatography (HPLC)

Methanolic extract of *B. ceiba* was fractionated with ethyl acetate. Briefly, 500 mg of methanolic extract was suspended in water (10 ml), fractionated with ethyl acetate (10 × 3 ml). The ethyl acetate was removed in vacuo to obtain 309.15 mg (61.83% yield) which was reconstituted with methanol and filtered through 0.22 μ nylon syringe filter. 10 μl samples were injected into the HPLC system for 50 min. Gallic acid was dissolved in HPLC-grade methanol. The HPLC system of Agilent technologies 1200 series was composed of EZ-chrome system controller, autosampler, LC-binary pump, diode array detector (at 254 nm) and Innoval C18 (4.6 mm × 250 mm) column. The mobile phase used for Gallic acid was 0.05% acetic acid in HPLC-grade water (component A) and 80:20 acetonitrile and 0.05% acetic acid (component B). The mobile phase was filtered through 0.45 μm membrane filter paper and sonicated. A gradient method was adopted as 0–10 min, 10% B; 10–20 min, 10–20% B; 20–30 min, 20–40% B; 30–40 min, 40–60% B; 40–45 min, 60–70% B; and 45–50 min, 70–10% B. The flow rate was set at 1.0 ml/min of the mobile phase.

### Quantification of Lupeol and β-sitosterol using high-performance thin-layer chromatography (HPTLC)

The quantification of Lupeol in petroleum ether and methanolic extract and β-sitosterol in petroleum ether extract of *B. ceiba* was performed using CAMAG HPTLC system which consists of an automatic Linomat V sample applicator, a chamber for developing TLC and a CAMAG TLC scanner for densitometric evaluation of chromatograms. CATS 4 software was used for interpretation of results. Petroleum ether extract was suspended in chloroform (10 mg/ml), the methanolic extract was in methanol (10 mg/ml), lupeol and β-sitosterol dissolved in chloroform (1 mg/ml). Silica gel 60 F254 HPTLC aluminum sheets [10X10 cm with 0.2 mm thickness] from E. Merck, USA was used. Plant extract sample (05 μl) in duplicate was applied over TLC plate 08 mm wide band using a sample applicator at 12 mm. Each standard marker: lupeol and β-sitosterol in different concentration (1–8 μg/ml) was applied for preparation of a calibration curve. Nitrogen gas was also supplied for drying of bands simultaneous. Band of each sample was developed in mobile phase n-hexane: ethyl acetate 8:2 in TLC developing camber, saturated with the mobile phase. The developed TLC plate was dried, derivatized with anisaldehyde sulphuric acid reagent and heated to 105 °C for 5 min. Scan the TLC plate densitometrically using TLC scanner 3 with software: WINCATS at 566 nm and a fingerprint profile photo was documented. The intensity of the sample in the chromatogram with *R*_*f*_ value was quantified at 566 nm [34, 35].

### In-vitro osteoblastic proliferation using UMR-106 cell lines

UMR-106 cells procured from National Centre for Cell Science (NCCS), Pune, India, were cultured in Dulbecco’s modified Eagle’s medium (DMEM) supplemented with 10% fetal bovine serum (FBS), penicillin 100 U/ml and streptomycin 100 mg/ml at 37 °C in a humidified atmosphere of 95% air and 5% CO_2_. At 80–90% confluence, cells were seeded in 96-well, at a density 2000 cells/ml, and allowed to attach for 24 h. Then the cells were washed using PBS and co-cultured with serum-free medium mixed with different concentrations of each test sample. The cells were then treated with petroleum ether and methanolic extracts of *B. ceiba* with varying concentrations: namely PE5, PE10 & PE15: petroleum ether extracts 5, 10, 15 μg/ml; ME5, ME10 & ME15: methanolic extracts 5, 10 and 15 μg/ml respectively; E2: estradiol (5 μg/ml) and vehicle for 48 h. 17β-Estradiol (E2) was used as a positive control. The medium was co-cultured for 48 h with the sample solutions in a humidified atmosphere of air 95% and CO_2_ 5%, then the medium was removed and added with MTT solution (1 mg MTT/ml PBS) into each well. The sample was incubated for another 4 h to allow MTT to metabolize to formazan. The supernatant: MTT [3-(4,5-dimethyl-thiazol-2-yl)- 2,5-diphenyl-tetrazolium bromide] was aspirated from the wells and DMSO (150 ml per well) was added to dissolve the formazan crystals formed. The absorbance was measured at a wavelength of 595 nm using an enzyme immunoassay plate reader and the percentage of cell proliferation was calculated using the following formula [36, 37].

$$ \%\mathrm{proliferation}=\left(\mathrm{OD}\ \mathrm{sample}-\mathrm{OD}\ \mathrm{control}\right)/\mathrm{OD}\ \mathrm{control}\times \kern0.37em 100 $$

### Alkaline phosphatase (ALP) activity

Cultured cells were rinsed with Ringer solution. Cells were lysed, and cellular material was transferred to buffer 250 μl (10 mM pH 7.5Tris HCl, 0.5 mM MgCl_2_ and 0.1% Triton X 100). The cellular material was homogenized using Teflon homogenizer. ALP activity was performed using p-nitrophenyl phosphate (4.34 mM) in buffer (100 mM glycine pH 10.3, 1 mM MgCl_2_. The mixture was incubated for 30 min at 37 °C. The enzymatic reaction was stopped mixing 50 μl of 1 M NaOH and the absorbance was measured at 405 nm [38].

### Acute toxicity study

The acute toxicity study of petroleum ether and the methanolic extract was carried out in 2000 and 5000 mg/kg in the present study. No adverse or toxic symptoms were observed under behavioral and biochemical assessments for 1st day, 2, 4 and up to 14 days and both the extracts were found to be safe at 2000 and 5000 mg/kg. The methanolic extract of stem bark of *B. ceiba* has previously been reported to be safe orally in acute toxicity study conducted by our research group and found effective against high fat-induced obesity at 100 and 200 mg/kg in rodents [39]. In the present study, the two dose levels: 100 and 200 mg/kg *p.o.* of both the extracts were identified to use in the further pharmacological evaluation.

### Procurement of animals and ethical consideration

Female Wistar albino rats weighing 180-240 g were procured from Animal house facility of Lala Lajpat Rai University of Veterinary Sciences, Hisar, Haryana, India and kept at the animal house establishment of Shoolini University, Solan, HP, India vide Reg. No. 1541/PO/a/11/CPCSEA. They were maintained at the temperature of 25 ± 2 °C and relative humidity of 45 ± 5% and provided with water and food ad libitum. The animals were initially acclimatized to experimental laboratory conditions for 7 days before to experimentation. The experimentation was duly approved from the Institutional Animal Ethical Committee vide protocol no. IAEC/SU-PHARM/13/017 and conducted in accordance with the guidelines of Committee for the purpose of control and supervision of experiments on animals (CPCSEA), New Delhi, India which is in confirmation with ARRIVE guidelines prescribed for in-vivo experimentation on small animals.

### Surgical ovariectomy-induced osteoporosis in female rat

Osteoporosis was induced by a surgical ovariectomy method as described by Gupta et al. [39, 40]. Female Wistar albino rats were employed and divided into eight groups, each comprising six animals (*n* = 6). Sample size in each group was determined by research papers relevant to the study. Animals were divided into different groups viz. group 1: NC served as normal control received vehicle; group 2: Sham served as surgically operated received vehicle; group 3: OVX control surgical ovariectomy treated received vehicle; group 4 & 5: PE100 & PE200 received petroleum ether extracts 100 & 200 mg/kg respectively; group 6 & 7: ME100 & ME200 received methanolic extract at 100 & 200 mg/kg respectively; and group 8: Std: Ralox received Raloxifene 1.0 mg/kg [41, 42]. All the drug treatments were suspended in a vehicle (1% tween 80), given *per oral* using oral gavage cannula after 5–6 days of recovery of surgical procedures for 28 days. Sham stands for ‘*placebo* surgery’, has been installed in the present study to compare the effect of surgical procedures/interventions only.

Rats were anesthetized using ketamine: xylazine at 10:80 mg/kg, *i.p.* and underwent a surgical removal of ovaries. Briefly, a 1–3 cm long midline dorsal incision was made in the abdomen region. The peritoneal cavity was assessed, and ovaries were located surrounding fat tissue. Blood vessels at the proximal end of fallopian tube were ligated with catgut stitches and ovaries were removed completely to ensure ovariectomy. Then interstitial muscles and skin were sutured, and postoperative care was done. During postoperative care, the animal was housed individually in a cage, an antibiotic powder was applied to wounds to prevent microbial infection and pus formation and the animal can recover for 3–5 days.

On completion of the protocol, blood samples were collected for biochemical estimations, the animals were sacrificed using a recommended method for euthanasia as per CPCSEA guidelines: decapitation and bones were isolated for biomechanical and histological assessments.

### Pharmacological assessments

#### Percentage body weight and BMD

The change in body weight (%) was assessed weekly. BMD was also assessed using the Archimedes principle. Left femur bone was hydrated in distilled water for 1 h. Bones were weighed, submerged in distilled water, then weighed out of the water. Density was calculated using the following formula:

$$ \mathrm{B}\mathrm{MD}=\left(\mathrm{A}/\mathrm{A}\hbox{-} \mathrm{B}\right)\times \mathrm{P} $$

Where A: weight of hydrated bone out of the water, B: weight of hydrated bone submerged in water, P: density of distilled water at a given temperature and A-B is the weight difference which is equivalent to the volume [43].

#### Estimation of serum ALP, calcium and phosphorus level

Serum biochemical estimations like alkaline phosphatase (ALP) using the Tris carbonate buffer method, calcium using O-cresophathaleincomplexone (OCPC) method and phosphorus using ammonium molybdate method were done using enzymatic kits from Erba Diagnostic Mannheim, Daman [44].

#### Estimation of serum estradiol level

Serum estradiol level was estimated by an electrochemiluminescence analyzer: automatic (Elecsys 1010; Roche Inc., Germany) using kit (100 T) and by enzyme immunoassay (EIA) using a spectrophotometric apparatus [45].

### Biomechanical assessments

#### Three-point bending of tibia

A supporter with two loading points, 13 mm apart from each other was used on the stage of the testing machine. The lateral surface of the tibia at the tibiofibular junction was placed upon the first point and proximal tibia upon the other. A rounded press head compressed the middle of the tibial shaft until a fracture occurred [46].

#### Compression of the fourth lumbar vertebra

The fourth lumbar vertebra was located and isolated. The fresh vertebra was placed on the flat metal stage and compressed until it fractured. The reading was recorded in Newtons [47].

#### Femoral neck loading test

In femoral neck loading test, the head was loaded with a parallel force to the shaft until fracture. A flat surface metal clamp was used to fix the proximal part of the femur perpendicular and to fix tight. Loading of head was done using a concave compression head (2.5 mm). The amount of force for breaking down head was noted [46].

#### Histological study of bone tissue

The femur bones of rats were embedded in 10% formalin solution. Bones were decalcified in 5% ethylenediaminetetraacetic acid. The decalcified bones were fixed in paraffin wax and cut into plane thin sections using manual microtome. Hematoxylin and eosin stains were used to examine the histopathological changes under a light microscope at 40X.

#### Statistical analysis

Research finding was presented as mean ± standard deviation (SD) and statistical analysis: one-way analysis of variance (ANOVA) was applied using GraphPad Prism software (version 5) followed by Bonferroni’s multiple comparison tests, a *post-hoc* test. A *p*-value< 0.05 was statistically significant.

## Results

The extraction of powdered *B. ceiba* Linn. stem bark resulted in a yield of petroleum ether extract 2.17% *w*/w and that of methanolic extract 6.37% w/w. Qualitative phytochemical screening of prepared extracts was presented in Table 1.

**Table 1:** Qualitative phytochemical screening of plant extracts of *B. ceiba*

S. No.	Chemical test	Pet. ether extract	Methanolic extract
1.	Alkaloids	-	–
2.	Anthraquinone glycosides	-	–
3.	Saponin glycosides	-	+
4.	Cardiac glycosides	-	–
5.	Carbohydrates	-	+
6.	Flavonoids	–	+
7.	Tannins	–	+
8.	Steroids	+	–
9.	Triterpenoids	+	+
10.	Fats/lipids/fixed oil	+	–
11.	Proteins and amino acids	–	–

(+) indicate presence and (−) indicate absence

### Quantification of Gallic acid in *B. ceiba* by HPLC

**Fig. 1:**
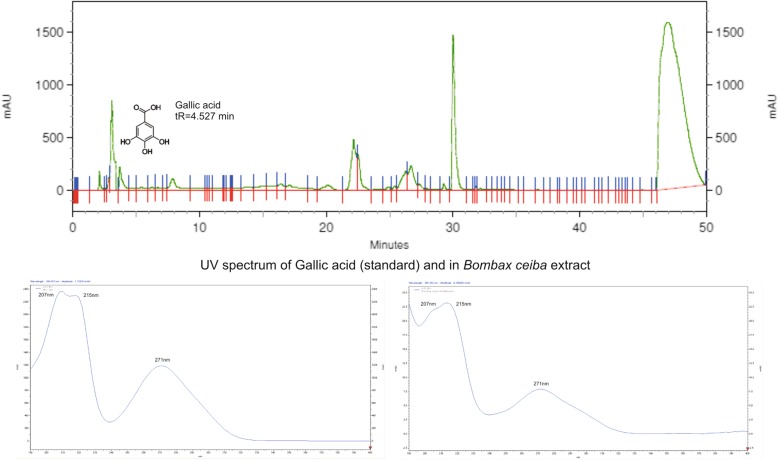
HPLC chromatogram for the quantification of gallic acid in the methanolic extract of *B. ceiba* and UV spectrum of Gallic acid at 254 nm

The HPLC chromatogram of Gallic acid was obtained at a wavelength of 254 nm. The HPLC chromatogram of a methanolic extract of *B. ceiba* extract was compared with the UV spectrum of Gallic acid in the test sample and presented in Fig. 1. A linear progression analysis with equation y = 63,432×-238,517, R^2^ = 0.9910 was carried out. Gallic acid in the stem bark of *B. ceiba* is being reported for the first time in the present study and was found to be 3.616% *w*/w in the methanolic extract (5.84% *w*/w in ethyl acetate extract of methanolic extract) with retention time 4.527 min.

### Quantification of β-sitosterol and lupeol in *B. ceiba* by HPTLC

**Fig. 2:**
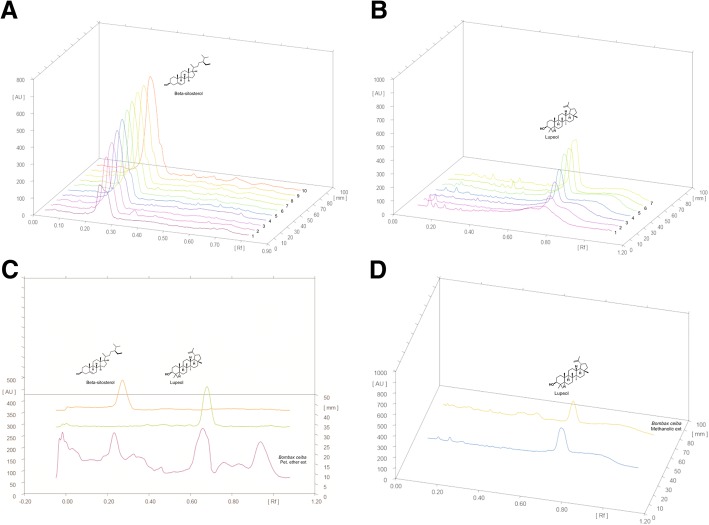
HPTLC chromatograms: **a** Calibration curve for β-sitosterol; **b** Calibration curve for lupeol; **c** Quantification for β-sitosterol and lupeol in petroleum ether extract; and **d** Quantification of lupeol in methanolic extract of *B. ceiba*. Results: mean ± SD, analyzed by one-way ANOVA followed by Bonferroni’s multiple comparison tests; ^*^P < 0.05 vs C (control); ^#^*P* < 0.05 vs PE15

A calibration curve of β-sitosterol and lupeol as concentration (μg/ml) vs area under the curve (AU) was plotted using HPTLC. A linear progression analysis for β-sitosterol with the equation: y = 1286.5× + 6096.6, R^2^ = 0.9562; and for lupeol with the equation: y = 1609.4 x-2662.4, R^2^ = 0.9901 were done. The percentage yield of β-sitosterol was 3.806% w/w with Rf value 0.27 in petroleum ether extract whereas of lupeol was 5.292% w/w in petroleum ether extract and 3.812% w/w with Rf value 0.67 in methanolic extract of *B. ceiba*. HPTLC chromatograms were presented in Fig. 2a, b, c and d.

### Osteoblastic cell proliferation and ALP activity

**Fig. 3:**
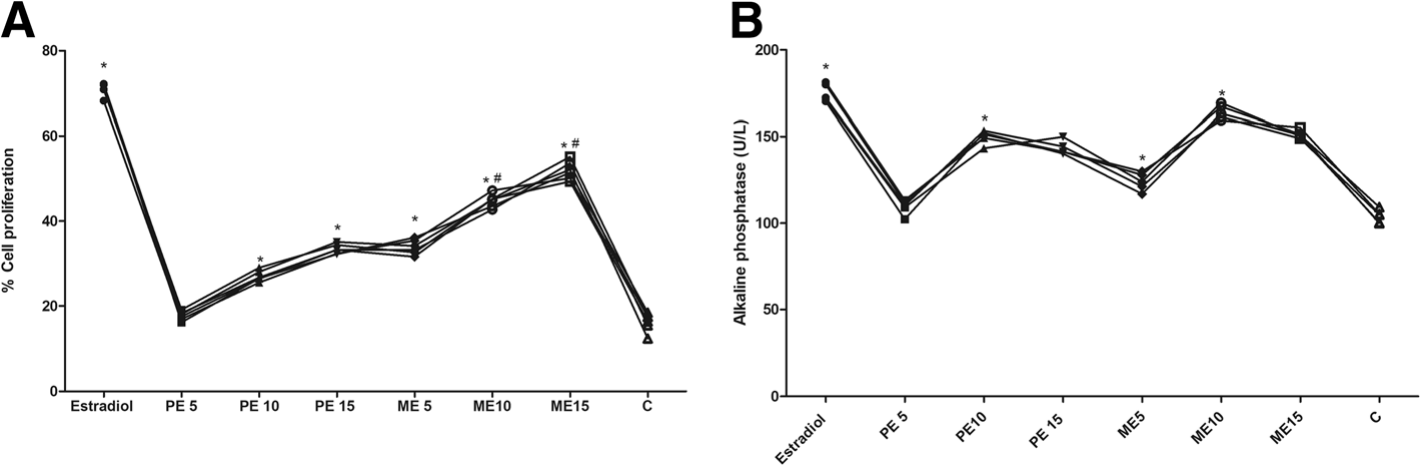
The effects of *B. cieba* on osteoblast-like UMR106 lines (**a**) Cell proliferation (**b**) ALP activity. Results: mean ± SD, analyzed by one-way ANOVA followed by Bonferroni’s multiple comparison tests; ^a^*p* < 0.05 vs. Sham; ^b^*p* < 0.05 vs. OVX; ^c^*p* > 0.05 vs. Std Ralox

The effect of ***B. ceiba*** extract on osteoblastic cell proliferation was studied by treatment of rat osteoblastic UMR-106 cells at concentrations of 5–20 μg/ml for 48 h. ***B. ceiba*** extract increased the optical density of the purple formazan product in the MTT assay. The results showed the greatest increase in cell proliferation at 10 μg/ml by both extracts. Whereas, the effect produced by methanolic extract at 10 and 15 μg/ml was significant to that of PE 15. Increase in cell viability and mitochondrial activity was estimated using MTT assay and increased osteogenic activity was also noted as detected by the ALP assay. However, the effect of ME15 and PE15 on ALP level UMR cells reflects the cessation of response till ME10 and PE10 concentrations respectively. The lower concentration of each extract from 0.5 to 5 μg/ml is suggested further for assessment over in-vitro assays (Fig. 3a and b).

### Effect of *B. ceiba* on the percentage change in body weight

**Fig. 4:**
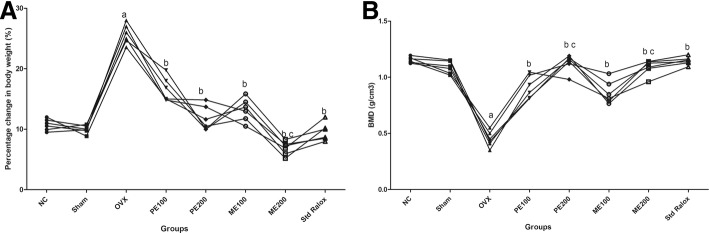
Effect of plant extracts of *B. ceiba* on (**a**) percentage change in body weight; and (**b**) bone mineral density. Results: mean ± SD, analyzed by one-way ANOVA followed by Bonferroni’s multiple comparison tests; ^a^*p* < 0.05 vs. Sham; ^b^*p* < 0.05 vs. OVX; ^c^*p* > 0.05 vs. Std Ralox

Surgical ovariectomy produced a significant increase in percentage change in body weight in OVX group, as compared to sham control. Petroleum ether and methanolic extracts at 100 and 200 mg/kg each of *B. ceiba* produced significantly (*p* < 0.05) decrease in percentage change in body weight as compared to OVX group. The effect of high dose (200 mg/kg) of methanolic extract was found to be insignificant (*p* > 0.05) to that of standard Raloxifene (Fig. 4a).

### Effect of *B. ceiba* on BMD

Surgical removal of ovaries caused a significant decrease in BMD in the OVX group as compared to sham control. Whether treated with a pet. Ether and methanolic extract at 100 and 200 mg/kg of *B. ceiba* produced significantly (*p* < 0.05) increase in BMD as compared to OVX group. In addition, the highest dose (200 mg/kg) of both the extracts produced insignificant (*p* > 0.05) increase in BMD to standard Raloxifene (Fig. 4b).

### Effect of *B. ceiba* on serum biochemical parameters

**Fig. 5:**
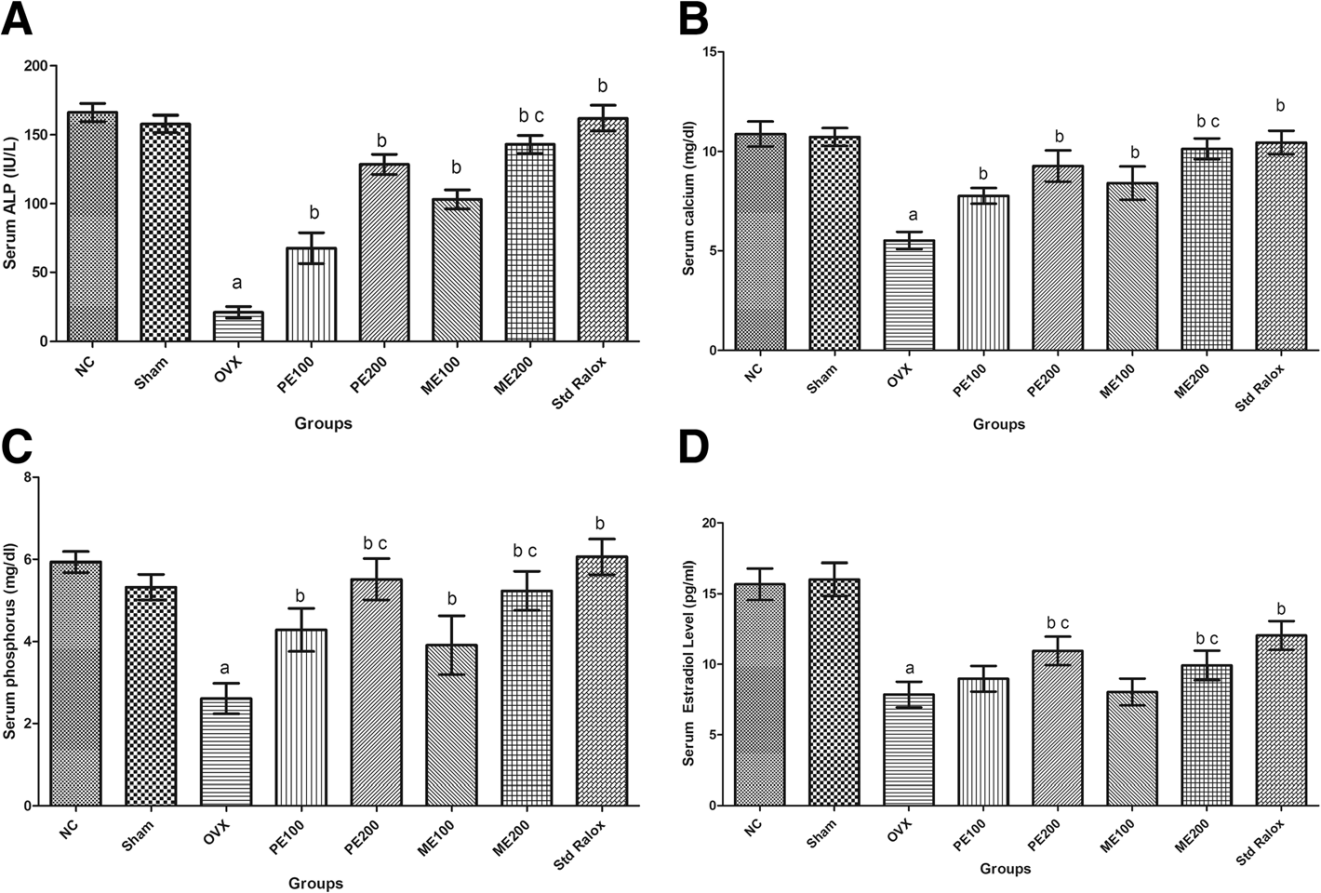
Effect of plant extracts of *B. ceiba* on serum biochemical parameters, (**a**): ALP, (**b**): calcium, (**c**): phosphorus, (**d**): Estradiol levels. Results: mean ± SD, analyzed by one-way ANOVA followed by Bonferroni’s multiple comparison tests;^a^*P* < 0.05 vs. Sham; ^b^*p* < 0.05 vs. OVX; ^c^*p* > 0.05 vs. Std Ralox

Surgical removal of both the ovaries produced significant (*p* < 0.05) reduction in serum ALP, calcium and phosphorus levels in the OVX control group, in comparison to sham control. Petroleum ether and methanolic extracts of *B. ceiba* at 100 and 200 mg/kg produced significant (*p* < 0.05) increase ovariectomy-induced serum biochemical changes, as compared to the OVX group. The effect produced by highest dose, 200 mg/kg of methanolic extract was found to be insignificant (*p* > 0.05) when compared with the standard drug: Raloxifene (Fig. 5a, b and c).

### Effect of *B. ceiba* on serum estradiol level

Deficiency of estrogen was noted due to surgical ovariectomy in female rats as compared to normal control (*p* < 0.05). Petroleum ether and methanolic extracts of *B. ceiba* were significantly recovered the estrogen level in comparison to OVX control (*p* < 0.05). Plant extract in highest doses could produce an effect equivalent to Raloxifene (*p* > 0.05) (Fig. 5d).

### Effect of *B. ceiba* on bone biomechanical parameters

**Fig. 6:**
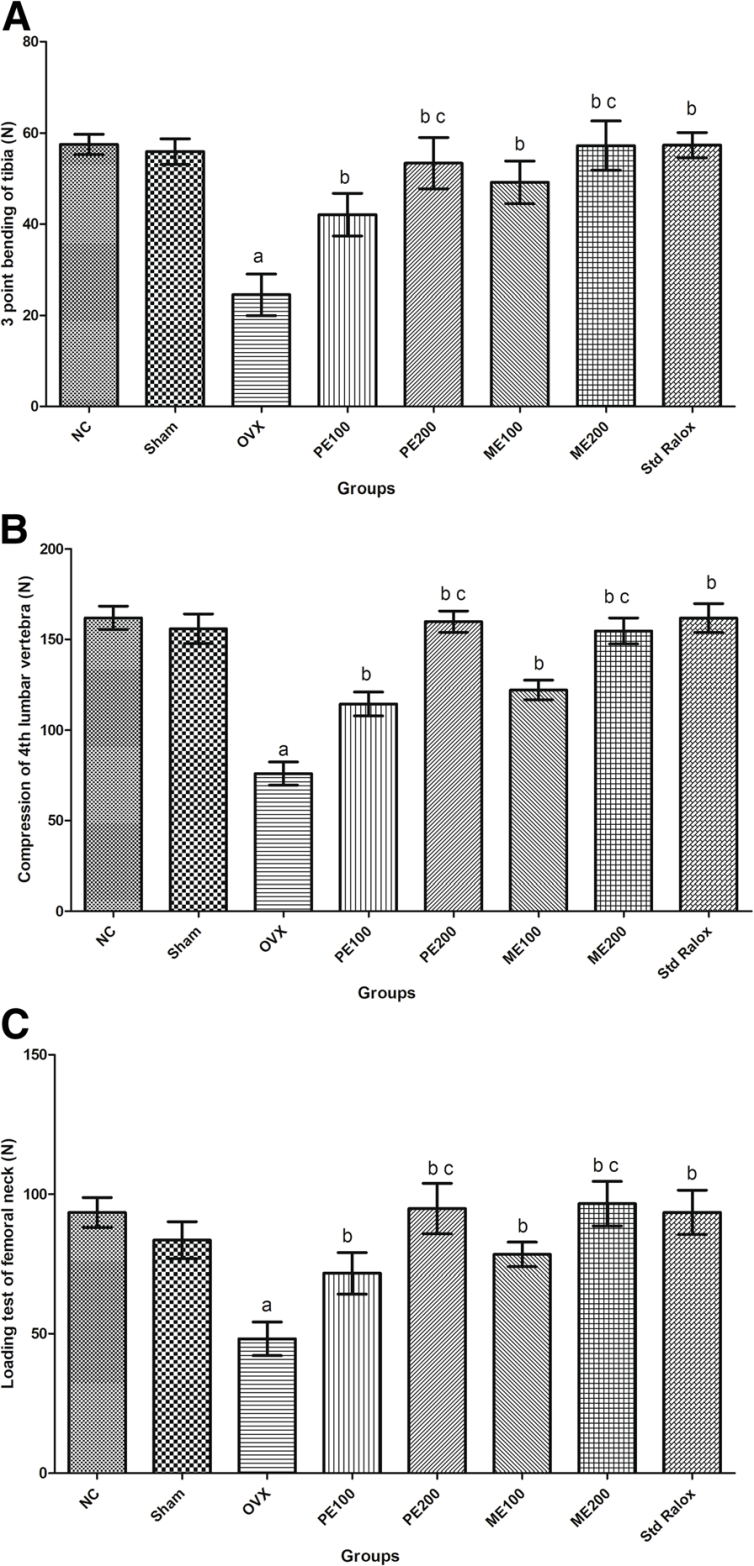
Effect of plant extracts of *B. ceiba* on biomechanical parameters; (**a**) Three-point bending of the tibia, (**b**) Compression of the 4th lumbar vertebra, (**c**) Loading test of femoral neck

In the OVX group, surgical ovariectomy produced a significant decrease in bone strength of tibia, lumber and femoral vertebra, as compared to sham control. On treatment with petroleum ether and methanolic extract of *B. ceiba* at 100 and 200 mg/kg caused significant (*p* < 0.05) increase in bone strength of the bones, when compared to OVX group. The highest dose, 200 mg/kg of both extracts produced an effect in strengthen insignificant (*p* > 0.05) increase in bone strength of the tibia as compared to standard Raloxifene (Fig. 6a, b and c).

### Effect of *B. ceiba* on bone histopathological characteristics

**Fig. 7:**
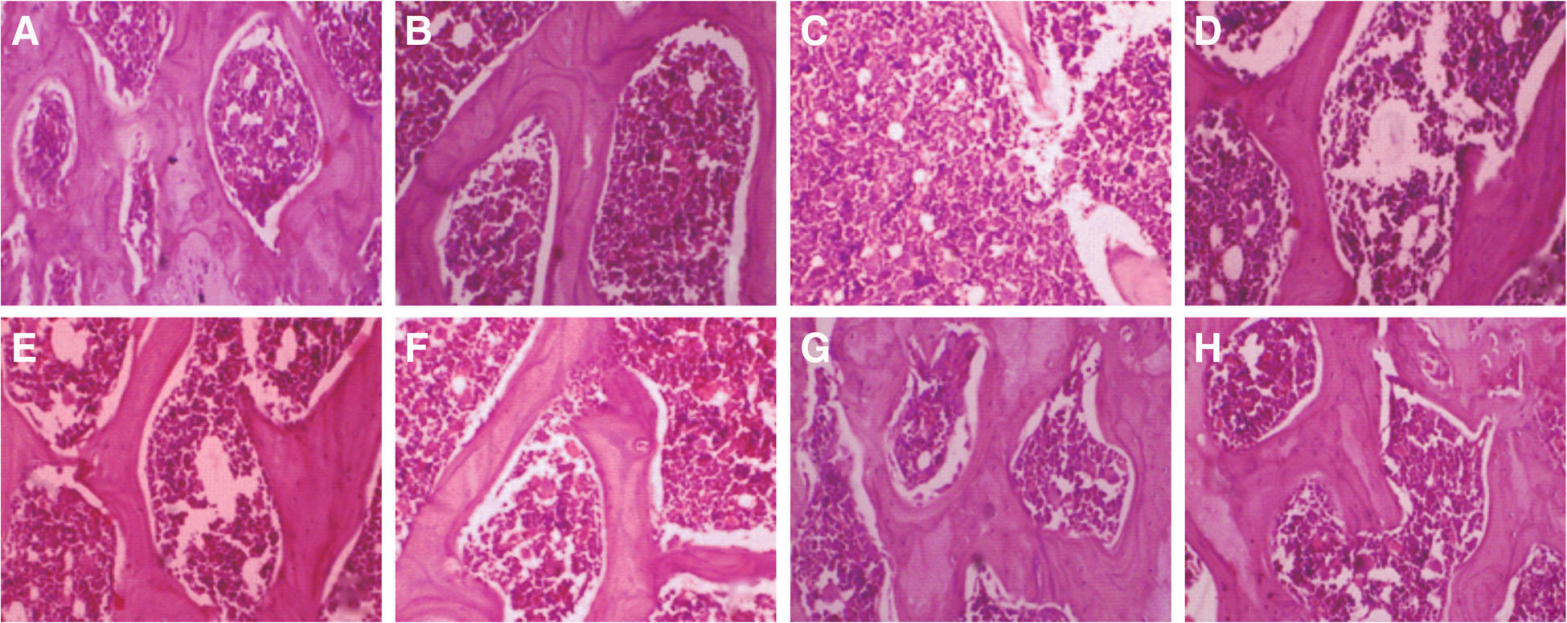
Effect of plant extracts of *B. ceiba* on histopathological parameters of rat femur; **a** and **b**: NC and Sham, Longitudinal section of femur of normal control group and sham control showing compact, normal and uniform trabecular bone; **c**: OVX, OVX group showed widely separated and thin bone trabeculae, loss of interconnectivity and widening of bone marrow spaces; **d** and **e**: PE100 and PE200, Petroleum ether extract (100 and 200 mg/kg) of *B. ceiba* showed less separated bone trabeculae, formation with reduced interconnectivity, narrowing of intertrabecular space; **f** and **g**:ME100 and ME200, Methanolic extract (100 and 200 mg/kg) of *B. ceiba* showed compact, normal, thick and uniform bone trabeculae, interconnectivity between bone trabeculae, narrowing of bone marrow spaces; **h**: Std Ralox, Standard drug: Raloxifene (1 mg/kg) showed thickening of trabecular bone and restoration of normal architecture of bone (10X)

The histopathological observations of right femur (proximal diaphysis region) of rats revealed compact, normal and uniform trabecular bone, which was recognized with appropriate interconnectivity between the bone trabeculae and narrow bone marrow spaces or inter-trabecular spaces in normal and sham control groups. OVX control group showed the destruction of the normal architecture of bone in the form of thinning of trabecular bone, loss of interconnectivity between trabeculae and widening of bone marrow spaces. Treatment with petroleum ether extract showed less separated bone trabeculae, interconnectivity between the bone trabeculae and reduced narrowing of bone marrow spaces. Methanolic extract of *B. ceiba* showed significant restoration of normal architecture as adequate interconnectivity between the bone trabeculae, narrowing of bone marrow spaces and thickening of trabecular bone. A significant restoration of bone integrity was also observed during treatment with standard drug Raloxifene (Fig. 7).

## Discussion

The present study evidenced for the anti-osteoporotic effect of the stem bark of *B. ceiba* by providing osteogenic, estrogenic effects and amelioration of bone remodeling in surgically ovariectomy-induced osteoporosis in the female rat. Postmenopausal osteoporosis occurs due to the ovarian deficiency of a hormone, estrogen, which is a most common cause of age-related postmenopausal bone porosity and fragility [48] and is possibly evident caused by a surgical ovariectomy method in female rats [49]. Osteoporosis occurs due to uncoupling between bone formation by osteoblasts and bone resorption by osteoclasts during the bone remodeling process [50]. Estrogen deficiency causes increased bone resorption, microarchitectural deterioration and reduced bone mass [51]. Estrogen is also reported to increase the activity of FAS [10] and FAS activation is reported to decrease the proliferation of osteoblast [14]. The release of an inflammatory mediator like TFG-β antagonizes the FAS activity [16]. Postmenopausal women become obese due to the accumulation of fat because estrogen helps in fat breakdown [52].

The results from in vitro study provide additional evidence for the protective effects of *B. ceiba* extract on the bone with enhancement of matrix ossification which was found in rat osteoblast-like UMR-106 cells indicating the existence of osteogenic activity of plant constituents. Plant extracts showed marked efficacy in the proliferation of UMR cell lines against MTT. Whereas in ALP expression study, the maximum effect was ceased at 10 μg/ml of each extract and highest concentration showed decreased response which may be due to saturation of target receptor sites.

The surgical ovariectomy in female rodents is a valid method to characterize the symptoms of osteoporosis and used to test the efficacy of new therapeutic agents [39, 40]. Surgical removal of both the ovaries in female rat causes decrease in estrogen level and the state is also attributed with an increase in body weight, decrease in bone mineral density, deterioration of bone integrity & architecture, increase in intertrabecular spaces or narrowing of bone marrow spaces as in ovariectomized animals. These changes resemble the clinical cases of increased bone fracture observed in postmenopausal women [53]. Treatment with petroleum ether and methanol extracts 100 and 200 mg/kg each of the *B. ceiba* and standard drug: Raloxifene (1 mg/kg) produced a significant decrease in percentage change in body weight and increase in BMD. The effect produced from the highest dose: 200 mg/kg of methanolic extract was as much as potent as that of standard drug. In the present study, surgical removal of both the ovaries caused a significant decrease in estrogen and its possible reversal is seen in treatment with a petroleum ether and methanolic extracts in high doses to some extent, as compared to OVX control. In women, estrogen is produced primarily from ovaries, corpus luteum, placenta and nongonadal organs such as liver, heart, skin adipose tissue and brain. In the postmenopausal state, the production and release of estrogen from non-gonadal organs appear to be vital for bone remodeling process by *B. ceiba* and maintains other body functioning.

Alkaline phosphatase is an enzyme found throughout the body and has been considered as a bone-specific marker for mineralization. ALP mainly represents bone metastases in a process of bone remodeling [54]. Ovariectomy results in decreased serum ALP level, which is an indication of bone loss [55]. Treatment with both the extracts *B. ceiba* produced a significant increase in serum ALP level. Calcium (Ca) and phosphate (P) are the two essential micronutrients and constituents of hydroxyapatite, the bone mineral which provides compactness and mechanical resistance to organic matrix. During postmenopausal state, the estrogen deficiency affects the absorption of the micronutrients from GIT and presence in the circulation which is maintained from the reservoir i.e. bone and causes a decline in the serum levels [56]. *B. ceiba* extract showed an elevated level of calcium which may be due to its effect on Ca absorption, and its maintenance in bone, activation of osteoanabolic and stimulation of gonadal effects contributing matrix deposition during osteogenesis [57]. In the present study, estrogen deficiency may cause the release of inflammatory mediators which further lead to activation of MHC-II and T cell producing more RANKL and TNF-α which are osteoclastogenic factors [58]. Estrogen deficiency causes an increase in FAS gene expression in the periosteum, negatively regulates the synthesis of TGF-β and BMP-2 in osteoblasts [17, 21] and results in a lipotoxic effect on osteoblasts. In the study, there was a significant decrease in bone strength of the tibia, femur and lumbar bones of OVX rat was observed. These changes observed in biomechanical assessments: three-point bending of the tibia, compression of the 4th lumbar vertebra, and loading test of the femoral neck, reflecting marker for reduced bone strength, enhanced bone fragility and deterioration [46, 47].

In the histological study, petroleum ether and methanolic extract at 100 and 200 mg/kg each of *B. ceiba* significantly reversed the changes of bone fragility or bone loss, as compared to the OVX group. This restorative effect of *B. ceiba* extracts is significantly emphasized by increased interconnectivity between the bone trabeculae and reduced narrowing of bone marrow spaces in the femur bone when compared with OVX control. The integrity and compactness of bone were also significantly lost with thin trabeculae and narrow bone marrow spaces, in comparison to normal and sham-treated rats as also confirmed by histological observations.

Qualitative phytochemical screening of methanolic extract showed the presence of saponins, carbohydrates, flavonoids, tannins, steroids, and triterpenoids which may be responsible to ameliorate oxidative and inflammatory interventions in bone disorders. HPLC analysis of methanolic extract revealed the presence of Gallic acid for the first-time in the present study. Gallic acid, a phenolic acid is found to improve the bone strength and reduces bone loss by providing antioxidant action. It is reported to inhibit osteoclastogenesis induced by RANKL in RAW 264.7 cell lines in-vitro [59]. Gallic acid is found in nuts, fruits, leaves (tea) as polyphenol and associated with antioxidant and anti-inflammatory activities [60, 61]. In another study at our laboratory, gallic acid has been lowered the surgically induced bone porosity and improved bone strength in female rats at 25 and 50 mg/kg. Lupeol has been quantified in petroleum ether and methanolic extracts using HPTLC study and identified as a major contributor for the efficacy of *B. ceiba* for bone porosity and bone loss. Lupeol is reported to inhibit osteoclast differentiation and bone resorption through RANK, nuclear factor kappa-B (NF-kB), nuclear factor of activated T-cells (NFATC) and c-Fos (a proto-oncogene) in-vitro and in-vivo in an animal model of hypercalcemia mediated bone porosity [62]. A low dose combination of lupeol acetate and curcumin prevents activated osteoclast precursor associated diseases such as rheumatoid arthritis and osteoporosis [63]. Lupeol may play a significant role in improving bone remodeling and thus correcting bone loss via the FAS gene and TGF-β. *B. ceiba* is also reported to have anti-inflammatory and antioxidant effects and decreases NO production [64, 65]. β-sitosterol, a phytosterol has been reported as an anti-inflammatory, angiogenic and hypocholesterolemic agent in in-vitro and in-vivo studies [66–68] and been quantified in the petroleum ether extract of the plant. In literature, *B. ceiba* is also documented to contain quercetin, a flavonoid which has been reported to lower loss of bone integrity cause due to ovariectomy in mice and restored bone repair [30, 31]. Flavonoid constituents are also identified qualitatively in *B. ceiba* methanolic extract under phytochemical screening which may have efficacy treating osteoporosis.

The efficacy of methanolic and petroleum ether extract was found to be similar in in-vitro and in-vivo studies. The presence of active constituents: beta-sitosterol (3.806% in petroleum ether extract), lupeol (5.292% petroleum ether extract and 3.812% in methanolic extract) and gallic acid (3.616% in methanolic extract) may be correlated with the efficacy of *B. ceiba* stem bark along with some other constituents: flavonoids and tannins (as confirmed by qualitative phytochemical screening). This finding may further signify the use of intact plant part for the disease.

## Conclusion

It may be concluded that *B. ceiba:* stem bark has pharmacological potential to ameliorate the condition of bone fragility and fracture due to the presence of phytoconstituents like containing lupeol, gallic acid and β-sitosterol. It can reverse the debilitating state of bone remodeling due to estrogenic modulation in-vitro and in-vivo and maintain calcium, phosphorus level, and bone integrity.
